# SlMYB75, an MYB-type transcription factor, promotes anthocyanin accumulation and enhances volatile aroma production in tomato fruits

**DOI:** 10.1038/s41438-018-0098-y

**Published:** 2019-02-01

**Authors:** Wei Jian, Haohao Cao, Shu Yuan, Yudong Liu, Juanfang Lu, Wang Lu, Ning Li, Jianhui Wang, Jian Zou, Ning Tang, Chan Xu, Yulin Cheng, Yanqiang Gao, Wanpeng Xi, Mondher Bouzayen, Zhengguo Li

**Affiliations:** 1 Key Laboratory of Plant Hormones and Development Regulation of Chongqing, School of Life Sciences, Chongqing University, 401331 Chongqing, China; 2 College of Resources, Sichuan Agricultural University, 611130 Chengdu, China; 3 College of Horticulture and Landscape Architecture, Southwest University, 400716 Chongqing, China; 4 Horticulture Institute, Sichuan Academy of Agricultural Sciences, 610066 Chengdu, China; 5 Institute of Special Plants, Chongqing University of Arts and Sciences, 402160 Yongchuan, China; 6 INRA, Genomique et Biotechnologie des Fruits, Chemin de Borde Rouge, F-31326 Castanet-Tolosan, France

**Keywords:** Secondary metabolism, Plant molecular biology, Plant breeding, Plant stress responses, Plant physiology

## Abstract

Genetic manipulation of genes to upregulate specific branches of metabolic pathways is a method that is commonly used to improve fruit quality. However, the use of a single gene to impact several metabolic pathways is difficult. Here, we show that overexpression of the single gene *SlMYB75* (*SlMYB75*-OE) is effective at improving multiple fruit quality traits. In these engineered fruits, the anthocyanin content reached 1.86 mg g^−1^ fresh weight at the red-ripe stage, and these *SlMYB75*-OE tomatoes displayed a series of physiological changes, including delayed ripening and increased ethylene production. In addition to anthocyanin, the total contents of phenolics, flavonoids and soluble solids in *SlMYB75*-OE fruits were enhanced by 2.6, 4, and 1.2 times, respectively, compared to those of wild-type (WT) fruits. Interestingly, a number of aroma volatiles, such as aldehyde, phenylpropanoid-derived and terpene volatiles, were significantly increased in *SlMYB75*-OE fruits, with some terpene volatiles showing more than 10 times higher levels than those in WT fruits. Consistent with the metabolic assessment, transcriptomic profiling indicated that the genes involved in the ethylene signaling, phenylpropanoid and isoprenoid pathways were greatly upregulated in *SlMYB75*-OE fruits. Yeast one-hybrid and transactivation assays revealed that SlMYB75 is able to directly bind to the MYBPLANT and MYBPZM *cis*-regulatory elements and to activate the promoters of the *LOXC*, *AADC2* and *TPS* genes. The identification of *SlMYB75* as a key regulator of fruit quality attributes through the transcriptional regulation of downstream genes involved in several metabolic pathways opens new avenues towards engineering fruits with a higher sensory and nutritional quality.

## Genetics: one small tweak, one giant leap for tomatoes

A single gene has been identified in tomatoes that regulates multiple aspects of fruit quality, including levels of health-promoting anthocyanins, opening the door to engineering more nutritious and better-tasting tomatoes. Fruit breeders have long-manipulated genes to enhance traits like yield or disease resistance, but it is rare to find a single gene that can improve multiple aspects of fruit quality. In this study, Zhengguo Li at Chongquing University in China and colleagues show that over-expressing a transcription factor called SIMYB75 results in striking deep purple tomatoes enriched in anthrocyanins–antioxidants thought to be protective against various diseases - which tomatoes are usually devoid of. They also had higher levels of other health-promoting phytochemicals and enhanced production of aroma volatiles, which can influence the taste and flavor of fruit.

## Introduction

As an indispensable daily supplier of nutrition to humans, fruit offers abundant nutrients, including vitamins, minerals, antioxidants and fiber^1^. Therefore, improving the nutritional and sensory qualities of fruit is becoming a primary goal to meet consumer requirements for healthier food. Nevertheless, breeding efforts to date have primarily concentrated on yield and disease resistance traits, whereas sensory and nutritional aspects such as the pigment, aroma, taste and health-promoting compounds have not received sufficient attention. In recent years, tremendous advances in functional genomics and biotechnology tools have presented new prospects for producing high-quality fruits that can better meet consumer expectations.

Anthocyanins are water-soluble pigments that are widely distributed in many plants, where they confer their typical appealing color to various tissues and organs in a number of species. With regards to their impact on human health, numerous studies have indicated that anthocyanins have high antioxidant properties, with the scavenging of active oxygen species^2^. In addition, anthocyanins have been reported to play a role in protecting against age-related degenerative diseases, especially by inhibiting tumor cell growth^3^. Anthocyanins are synthesized via the flavonoid pathway, which is a branch of the phenylalanine pathway^4^. The accumulation of anthocyanins in plants is regulated by a series of environmental conditions and developmental signals^5,6^. Two types of genes have been suggested to participate in the anthocyanin pathway, (i) those encoding key enzymes for anthocyanin biosynthesis^4^ and (ii) those coding for the transcription factors (TFs) that regulate the spatio-temporal expression of functional genes^7^. Three types of TFs, R2R3-MYB, bHLH and WD40, are known to control the anthocyanin biosynthetic pathway, and most likely, these TFs work together through the formation of a transcription complex named MBW^8,9^. However, several studies have indicated that MYB TFs could also function independently of the MBW complex to regulate the biosynthesis of anthocyanins, thus assigning a crucial role to MYB factors *per se* in controlling anthocyanin biosynthesis^7,10,11^.

Tomatoes are one of the most widely consumed fruit crops in the world, which motivates their frequent use as a model species to study nutritional metabolism. Moreover, the tomato is an excellent candidate for the transgenic enhancement of its flavonoid content because of its low basal content of these compounds^3,12^. Although tomato plants with both *Aft* and *atv* alleles could accumulate small amounts of anthocyanin, the mechanism and key genes controlling this variation have not yet been deciphered^13,14^. The silencing of the *SlDET1* gene in tomatoes has resulted in altered light-mediated developmental processes along with an enhanced flavonoid content^15^. Similarly, overexpression of the chalcone isomerase gene in tomatoes could increase the flavonoid content up to 78-fold exclusively in the peel, but the overall level in the fruits remained low^12^. In contrast to the functional genes, manipulating the expression of TFs proved more effective at modulating the secondary metabolism. Lc and C1 are two TFs in maize that control anthocyanin biosynthesis; however, overexpression of these two TFs in tomatoes greatly increased the flavonoid content, but not the anthocyanin content^16^. Overexpression of *AtMYB12* under the control of the fruit-specific *E8* promoter resulted in orange tomatoes that contained significantly high contents of flavonoid and caffeoyl quinic acid^17^. However, the most significant increase in the anthocyanin content of tomato fruits was first obtained by expressing *Del* and *Ros1*, two snapdragon genes encoding bHLH and MYB TFs, respectively, which were under the control of the *E8* promoter, resulting in fully purple tomato fruits^3^. More recently, crossing the *AtMYB12*-overexpression line with the *Del/Ros1* line was reported to enhance anthocyanin accumulation^18^. Interestingly, this study indicated that *AtMYB12* not only promoted flavonoid biosynthesis, but it also improved the carbon supply from the primary metabolism, energy and reducing power, which resulted in a larger aromatic amino acid supply for secondary metabolism^18^. It is important to mention that the purple tomato fruits obtained in these studies always resulted from the cooperative action of two or more TFs. This result causes difficulty for traditional breeders, since it is challenging to find a wild species that could highly express these two or three key regulatory genes at the same time.

Recently, the *MYB* TF gene *Cs6g17570* was identified to play a critical role in regulating anthocyanin biosynthesis in blood oranges^19^. Interestingly, the upregulation of its homologous gene *AtMYB113-like* resulted in the increased accumulation of anthocyanin in *Arabidopsis*, whereas its downregulation led to a reduced amount of this pigment. Moreover, this process was reported to be dependent on the MBW complex^20^. Through a homology alignment, we identified a tomato gene, named *SlMYB75* (SGN, https://solgenomics.net/search/locus) or *SlAN2*, which showed the highest sequence similarity with *Cs6g17570* (Supplementary Fig. S1). Overexpression of *SlAN2* was previously reported to improve tomato plant resistance to high temperature, cold and oxidative stresses^21,22^. A recent study showed that overexpression of *SlANT1* or *SlAN2* induced accumulation of anthocyanin that was unevenly distributed in tomato fruit, but only *SlAN2* was able to act as a positive regulator of anthocyanin biosynthesis in vegetative tomato tissues under stress conditions^23^. At the same time, *SlAN2-overexpression* fruits displayed an orange color, fast softening and higher ethylene content^24^. In addition, several other MYB TFs are also involved in some fruit quality attributes, such as primary metabolism, secondary metabolism and organic acid metabolism^18,25^, and a consumer sensory analysis showed that there is a consumer preference for purple tomato fruits, for their better perceived flavor^26^. Thus, the potential role of *SlMYB75* in tomato fruit quality remains to be investigated.

In the present study, we attempt to describe the physiological and metabolic changes in purple tomatoes produced by overexpressing a single SlMYB75 TF (*SlMYB75*-OE). The data indicated that *SlMYB75* can effectively induce the accumulation of anthocyanin in various tissues, and its ectopic expression leads to increased ethylene production and enhanced phenolic, flavonoid and volatile aroma contents. Moreover, transcriptomic profiling showed that many genes involved in the ethylene response, phenylpropanoid and isoprenoid pathways were greatly affected in *SlMYB75*-OE fruits. Using yeast one-hybrid and dual-luciferase assays, we found that the conserved MYBPLANT (AAACCAACCC) and MYBPZM (ACCTACCC) elements were the core binding sites of SlMYB75, and we revealed the ability of SlMYB75 to activate the promoters of the *LOXC*, *AADC2* and *TPS* genes. This study demonstrates that a single SlMYB75 TF is able to increase the anthocyanin content to 1.86 mg g^−1^ fresh weight at the red ripening stage and promote volatile aroma accumulation partly through the transcriptional regulation of downstream genes involved in the corresponding metabolic pathways.

## Materials and methods

### Plant materials, growth conditions and chemicals

*Solanum lycopersicum* cv. Micro-Tom was selected as the wild type (WT) in this study. All the tomato seedlings were grown in an intelligent greenhouse under standard conditions (16/8 h and 25 °C/18 °C day/night cycle, 80% humidity and 250 µmol m^−2^ s^−1^ light intensity). For the hormone and stress treatments, the leaves of 1-month-old tomato plants treated with different reagents were harvested. For each tissue/organ type, samples were collected from at least six healthy plants. All the samples were mixed and frozen under liquid nitrogen immediately. The sample experiments were conducted three independent times.

For the chemicals, Folin-Ciocalteu phenol reagent was purchased from Sigma (St. Louis, MO, USA). HPLC-grade methanol was purchased from Merck KgaA (Darmstadt, Germany). The other reagents were all of analytical grade and were purchased from Sangon Biotechnology Co., Ltd. (Shanghai, China).

### Vector construction and plant transformation

The full length of the *SlMYB75* coding sequence was amplified from tomato cDNA and then cloned into the K303 expression vector, which contain two *35s* promoters from Gateway technology. The final vector was transferred into GV3101, and *Agrobacterium*-mediated transformation was performed as described previously^27,28^. The positive transgenic plants were identified by PCR, and homozygous plants from T2 or later generations were used for the experiments. All the primers used in this study are listed in Supplementary Table S2.

### RNA-Seq analysis

Total RNA was extracted from the fruits at the mature green (MG) and breaker (BR + 0) stages using an RNeasy Plant Mini Kit (Tiangen, China) according to the manufacturer’s protocol. Samples from both WT and *SlMYB75*-OE fruits were collected with two biological replicates. The concentration and quality of the RNA were assayed using a NanoDrop Lite spectrophotometer (Thermo Scientific). The cDNA libraries were constructed and then sequenced using a BGISEQ-500 System (BGI Inc.). Clean data were obtained after they were filtered and then mapped to the reference genome of *S. lycopersicum* in the Tomato SGN database (http://solgenomics.net/) with *Bowtie2*. The homogenized data were used to calculate the gene expression levels with *RSEM*. The criteria for defining differentially expressed genes (DEGs) were fold change ≥2.00 and *P* value ≤ 0.05. The raw transcriptome reads of this study have been deposited in the NCBI Short Read Archive under accession number SRP158557.

### Real time quantitative PCR

One microgram of total RNA (RNeasy Plant Mini Kit, Tiangen) was used to synthesize first strand cDNA with PrimeScript^TM^ RT reagent Kit using gDNA Eraser (Perfect Real Time) (TAKARA, Japan). Quantitative real-time PCR was performed with a Bio-Rad CFX system (Bio-Rad, USA) using SYBR^®^ Premix Ex Taq^TM^ (Tli RNaseH Plus) (TAKARA, Japan). Each sample was collected as three independent biological replicates, and the relative fold differences were calculated using a comparative Ct method. *SlUBI* was used as the internal reference over the entire experiment.

### Measurement of metabolites

For the total anthocyanin extraction, 0.1 g of lyophilized powder samples was incubated in 1 mL of pH 1.0 buffer solution (50 mM KCL, 150 mM HCL) and 1 mL of pH 4.5 buffer solution (400 mM CH_3_COONa, 240 mM HCL) for 24 h in the dark at 4 °C with gentle shaking, respectively. The samples were centrifuged at 14,000×*g* for 20 min. The total anthocyanin content in the supernatant was measured spectrophotometrically and expressed in mg of petunidin-3-(p-coumaroyl rutinoside)-5-glucoside (extinction coefficient 17000, molecular weight 934) per gram fresh weight^3,23^.

For the ethylene measurement, fruits at different development stages were harvested and placed in an open 50 mL jar for 2 h to minimize the wound ethylene caused by picking. The jars were sealed and incubated at room temperature for 1 h, and 1 mL of headspace gas was collected and then injected into a VARIAN CP3800 gas chromatograph equipped with a flame ionization detector (USA). The samples were compared with reagent-grade ethylene standards of known concentrations and normalized to the fruit weight^29^. At least 10 individual fruits were measured for each sample.

The total soluble solids and titratable acids were determined using a digital refractometer (Atago PR-101R, Tokyo, Japan) and titration method, respectively^30^. Each replicate contained 20 fresh tomato fruits and all the determinations were performed in triplicate.

For the total phenolic and flavonoid extractions, 1 g of lyophilized powder samples were incubated with methanol (80%, 24 mL) at 25 °C for 12 h with shaking and then centrifuged at 3000×*g* for 10 min at 4 °C. The residue was extracted twice more using the same procedure, and then the supernatants were collected and finally diluted to 50 mL with methanol to detect the total phenolics and flavonoids. The total phenolic content was determined using the Folin-Ciocalteu method. The total flavonoid content was measured as described previously^31^.

To measure the primary component of phenolics and flavonoids, 0.5 g of lyophilized powder was extracted with methanol (80%, 12 mL) and dimethyl sulfoxide (1:1, v/v) using the same procedures as the procedure used with the total phenolics and flavonoids. After the samples were filtered through a 0.22 μm syringe filter, the phenolics and flavonoids were measured using the HPLC method described previously^31^.

The concentration of aroma volatiles was determined as described previously, with some modifications^30^. Three grams of lyophilized powder were homogenized with 5 mL of saturated sodium chloride solution, and then 5 μL of ethyl nonanoate was added as an internal reference. The solution was incubated at 40 °C for 30 min, and a solid-phase microextraction (SPME) needle with a 1 cm-long fiber coated with 65 μm divinylbenzene/carboxen/polydimethylsiloxane (DVB/CAR/PDMS) fibers (Supelco Co., Bellefonte PA, USA) was used to extract the volatiles. A GCMS-QP2010 gas chromatograph-mass spectrometer system (Shimadzu Corporation, Kyoto, Japan) with an Rtx-5MS (Restek)-fused silica capillary column (5% diphenyl, 95% dimethyl polysiloxane) (0.32 mm, 30 m, 0.5 lm, J&W Scientific, Folsom CA, USA) was used for the compound confirmation. GC-MS Postrun Analysis software (SHIMADZU, GC-MS-QP2010, Japan) was used to evaluate the chromatograms and mass spectra. The compounds were identified by comparing their mass spectra with the data system library (NIST08). The concentrations of the volatile components were expressed as ng g^−1^ FW h^−1^.

### Yeast one-hybrid assay

Yeast one-hybrid (Y1H) assays were performed using a Matchmaker Gold Yeast One Hybrid System (Clontech). To construct the prey and bait vectors, the full length of the *SlMYB75* open reading frame (ORF) sequence was cloned and inserted into the pGADT7 vector, and the eight conserved *cis*-elements were cloned into the pAbAi vector. The bait plasmids were transformed into the Y1H Gold strain according to the manufacturer’s instructions. Aureobasidin A (AbA) was used to screen the minimal inhibitory concentration for the bait strains. The prey plasmid was transformed into a bait yeast strain to determine the DNA-protein interaction by screening them on SD medium with AbA and without leucine.

### Dual-luciferase transient expression assay

For the dual-luciferase assay, the full-length ORF of *SlMYB75* was cloned and inserted into a pGreenII 62-SK vector (effector), and the promoter sequence of the specific different expression gene (DEG) was cloned and inserted into pGreenII 0800 LUC vector (reporter). After their transformation into GV3101, the effector and reporter strains were cultured and then resuspended with infiltration buffer (10 mM MES, 10 mM MgCl_2_, 200 mM acetosyringone, pH 5.6) to an OD 600 of 1.0–1.5. The mixtures of effector and reporter cultures were infiltrated into *Nicotiana benthamiana* leaves with needleless syringes. Firefly luciferase and *Renilla* luciferase were assayed at 3 days after infiltration using Dual Luciferase Reporter Assay System reagents (Promega). The binding activity of SlMYB75 to the promoter of each specific DEG was calculated by finding the LUC to REN ratio. At least six biological replicates were conducted for each combination^32^.

### Statistical analysis

All the experiments were repeated at least three times, and the results were presented with the standard deviations. Student’s *t-*tests were used to analyze the data, and a difference was considered to be statistically significant when *P* *<* 0.05.

## Results

### Overexpression of a single *SlMYB75* induces anthocyanin accumulation

**Fig. 1:**
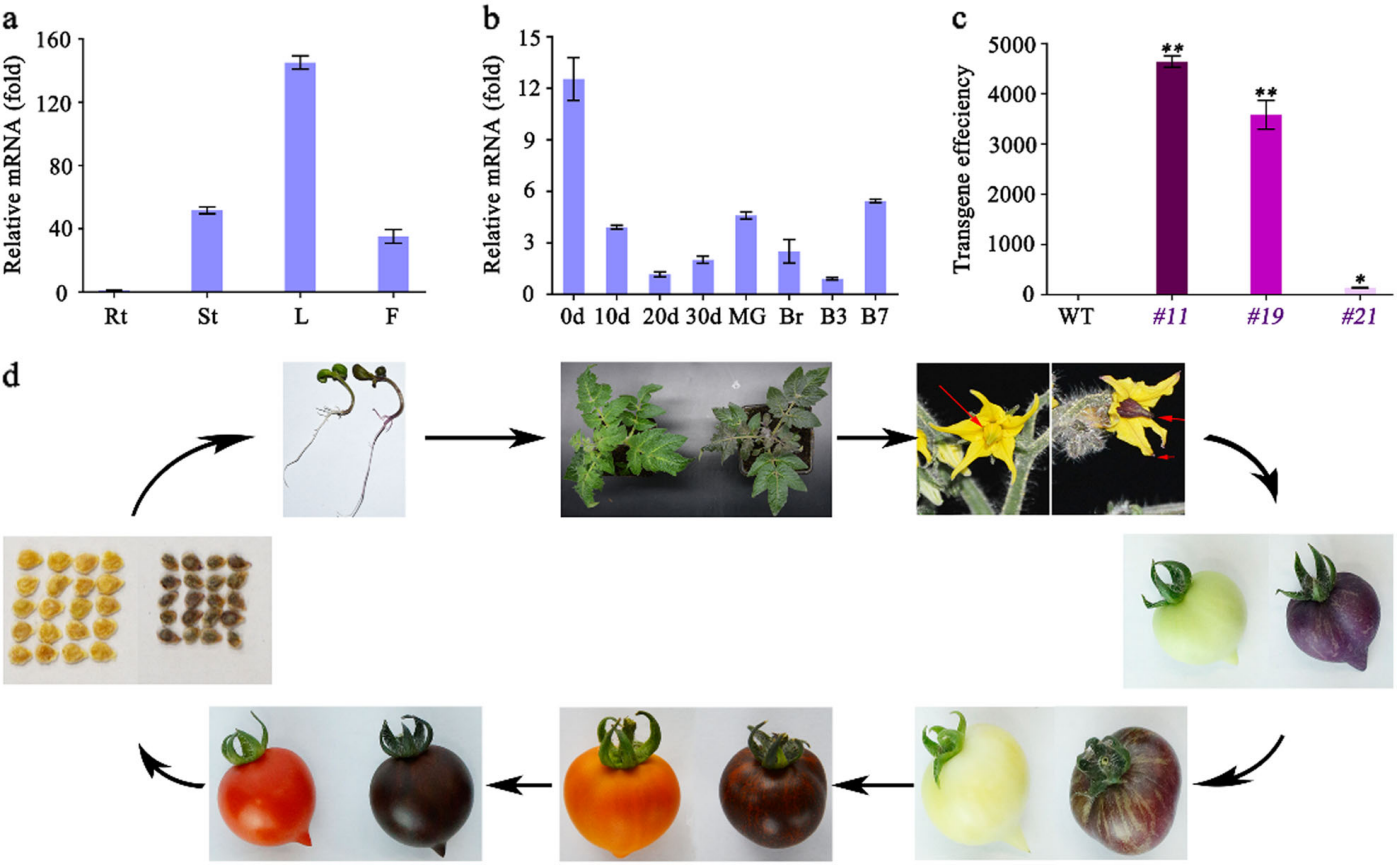
*SlMYB75* expression pattern and generation of transgenic plants. **a**, **b**, qRT-PCR analysis of *SlMYB75* expression level. Rt, root; St, stem; L, leaf; and F, flower; 0, 10, 20, and 30 d represent the fruits at 0, 10, 20, and 30 d after flowering; MG, mature green; and Br, B3, and B7 represent the fruits at 0, 3, and 7 d after the breaker stage. **c**, The *SlMYB75* mRNA level in fruits from the *#11*, *#19* and *#21* transgenic lines. **d**, Phenotypic characterization of WT (left) and *#11* (right) plants at different growth stages. The data represent the means ± SD of three biological replicates. “*” and “**” indicate significant differences between *SlMYB75*-OE tomato and WT with *P* < 0.05 and *P* < 0.01, respectively, as determined by the t-test

A qRT-PCR investigation of the *SlMYB75* expression pattern indicated that transcripts corresponding to this gene are detected in all tissues, but they display higher expression levels in vegetative tissues, especially in leaf organs (Fig. 1a and b). However, unlike the *Cs6g17570* gene^19^, the expression level of *SlMYB75* in tomato fruit organs is very low (Fig. 1a, b). To address the functional significance of *SlMYB75*, a tomato “Micro-Tom” cultivar was transformed with a sense construct of *SlMYB75*, leading to the generation of three independent transgenic homozygous lines. The expression of *SlMYB75* in *SlMYB75*-OE plants was assessed by *q*RT-PCR, indicating a dramatic upregulation in line *#11* and line *#19*, while the *#21* line only showed a slight upregulation (Fig. 1c), and then lines *#11* and *19* were selected to perform further experiments. A phenotypic evaluation revealed that the *SlMYB75*-OE lines accumulated abundant amounts of anthocyanins in both vegetative and reproductive organs, especially in the stamen, in which the color turned totally purple (Fig. 1d). Furthermore, the *SlMYB75*-OE lines displayed significantly smaller seeds than the WT (Fig. 1d). To further investigate the expression characteristics of *SlMYB75*, we checked the expression level of this TF in WT seedlings treated with different hormones or ones that were subjected to stress conditions. The data showed that *SlMYB75* is responsive to all the treatments applied here, consistent with the presence of the corresponding *cis*-elements identified in the promoter of *SlMYB75* (Supplementary Fig. S2). These results indicated that overexpressing a single SlMYB75 TF can lead to abundant anthocyanin accumulation in both vegetative and reproductive organs, and this TF could be induced by various hormones or stress conditions.

### Physiological features of the *SlMYB75*-OE tomato plants

**Fig. 2:**
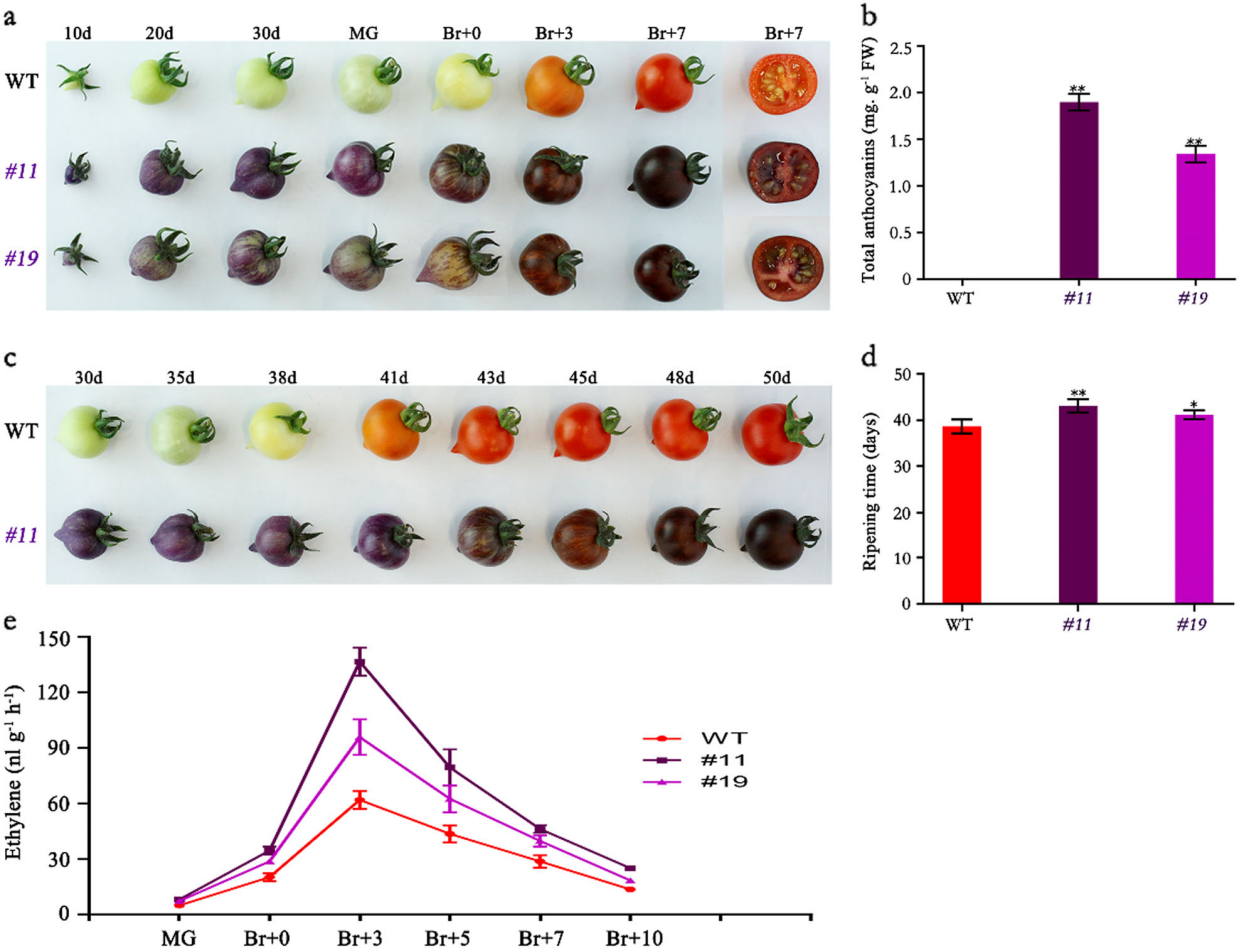
Phenotypic and physiological characteristics of WT and SlMYB75-OE plants. **a**, Phenotype of WT, *#11* and *#19* fruits at different developmental stages. **b**, The total anthocyanin content was measured in WT and *SlMYB75*-OE fruits at BR + 7. **c**, Fruit ripening process of a representative line (*#11*) compared with the WT. **d**, Quantification of fruit ripening by days to reach the breaker stage in the WT and *SlMYB75*-OE lines. The first and second branches of each plant from a total of 20–24 plants for each line were used to quantify the ripening time. **e**, Ethylene production of WT and *SlMYB75*-OE fruits was measured at different developmental stages. The values represent the means of at least 10 individual fruits. The statistical significance was based on Student’s *t*-test, *P < 0.05; and **P < 0.01, and the data are the means ± SD

One of the striking features of the *SlMYB75*-OE lines is the purple color exhibited by several organs, including the seeds, stamens and fruits. An assessment of the anthocyanin accumulation revealed high levels in the *SlMYB75*-OE fruits, and these compounds were not detected in the WT (Fig. 2a, b). Despite their deep purple color, it was easy to identify the different ripening stages of the transgenic fruits by checking the color of the low-anthocyanin region (Fig. 2a). Moreover, *SlMYB75*-OE plants exhibited 3–5-day delays in the occurrence of fruit ripening onset compared to the WT (Fig. 2c, d). Strikingly, ripening-associated ethylene production was significantly higher in *SlMYB75*-OE tomatoes at all ripening stages (Fig. 2e), which contrasted with the delayed ripening. These data suggested that overexpression of a single SlMYB75 TF can result in a series of physiological changes.

### Transcriptome profiling of the *SlMYB75*-OE tomato fruits

**Fig. 3:**
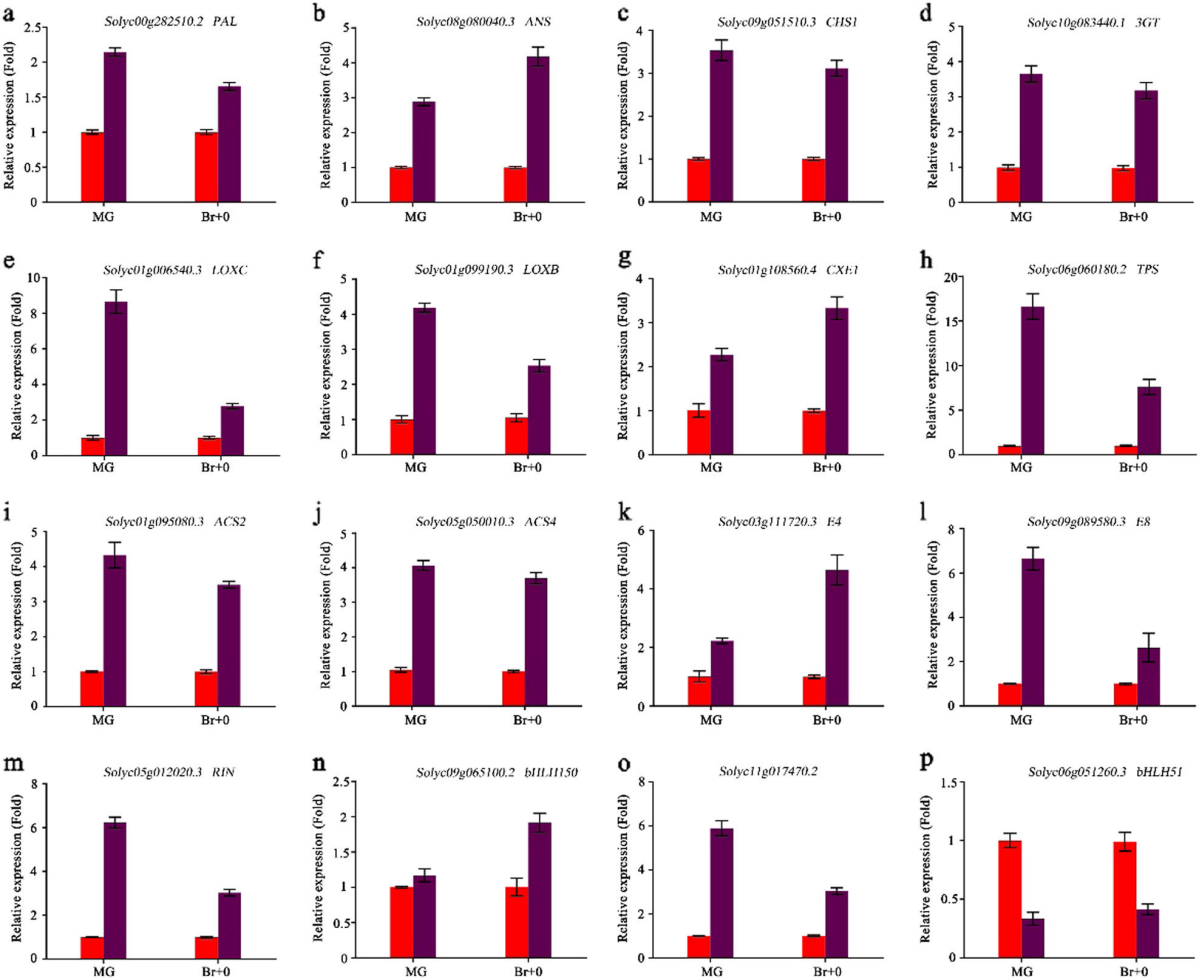
Genes selected from the transcriptome data were validated by qPCR. Four phenylalanine metabolism-related, four aroma volatile-related, five ethylene signaling-related and three transcription factors were selected. The red column represents WT fruits, and the purple column represents *SlMYB75*-OE fruits. The data represent the means ± SD of three biological replicates

To gain insight into the extent of transcriptomic reprogramming induced by the ectopic expression of *SlMYB75*, we performed genome-wide transcriptomic profiling in WT and *SlMYB75*-OE fruits (line *#11*) at the MG and BR + 0 stages. Compared to the WT, a total of 1576 and 2843 genes were differently expressed (DEGs) in *SlMYB75*-OE tomatoes at the MG and BR + 0 stages, respectively (Files S1 and S2). Based on the Gene Ontology (GO) assignment, the common DEGs of the MG and BR + 0 stages (675 common DEGs) were assigned to three categories, namely, biological process, cellular component and molecular function. “Biological process” was primarily focused on metabolic and cellular processes. Alterations in the molecular function analysis were primarily involved in the binding and catalytic activities (Supplementary Fig. S3 and File S3). Furthermore, 675 common DEGs were enriched in 73 KEGG pathways. The top five enriched pathways were the biosynthesis of secondary metabolites, phenylpropanoid biosynthesis, protein processing in the endoplasmic reticulum, plant hormone signal transduction and flavonoid biosynthesis (Supplementary Fig. S4 and File S4). These results indicated that *SlMYB75* overexpression impacted multiple processes, including transcription, stress responses, secondary metabolism and phytohormone signaling pathways. Based on the phenotype properties and potential application values of the *SlMYB75*-OE tomato, we focused on genes involved in metabolic processes, ripening-related processes and TFs (Table 1). To validate the accuracy of the generated transcriptome data, a total of 16 genes related to the above-cited processes were selected for qRT-PCR analysis. The outcome of this targeted expression analysis was highly consistent with the transcriptome data for all the tested genes (Fig. 3).

**Table 1:** List of different expression genes (DEGs) between WT and *#11* tomato fruits at the MG and BR + 0 stages. Genes marked with asterisks were validated by qPCR

ITAG 2.40 Tomato	Log2-fold (#11/WT; MG)	Log2-fold (#11/WT; BR + 0)	Functional annotation
Phenylalanine pathway			
Solyc10g086180.2	1.66	1.07	Phenylalanine ammonia-lyase
Solyc00g282510.2*	1.68	1.03	Phenylalanine ammonia-lyase-like
Solyc05g052240.3	1.34	1.71	Probable chalcone-flavonone isomerase 3
Solyc08g080040.3*	1.51	1.68	Leucoanthocyanidin dioxygenase-like
Solyc09g091510.3*	1.87	1.60	Chalcone synthase 1
Solyc12g088170.2	8.96	10.01	Anthocyanin acyltransferase
Solyc10g083440.1*	1.85	1.46	Anthocyanidin 3-O-glucosyltransferase-like
Flavor related			
Solyc01g006540.3*	2.86	1.19	LOXC
Solyc01g099190.3*	2.49	1.46	LOXB
Solyc01g006560.3	2.25	1.38	LOXF
Solyc08g006740.3	2.00	1.71	AADC2
Solyc01g108560.4*	1.03	2.16	CXE1
Solyc06g060180.2*	4.40	1.90	TPS
Solyc10g011920.2	1.10	1.66	Aromatic amino acid lyase
Ripening related pathway			
Solyc01g095080.3*	2.20	1.94	ACS2
Solyc05g050010.3*	1.80	1.98	ACS4
Solyc03g111720.3*	1.91	3.69	E4
Solyc09g089580.3*	1.74	1.50	E8
Solyc09g075440.3	1.23	1.76	ETR3(NR)
Solyc05g012020.3*	3.09	1.68	RIN
Solyc03g044300.3	2.39	1.40	AP2a
Solyc03g031860.3	2.60	1.71	PSY1
Solyc10g080210.2	2.54	2.33	PG2a
Other genes			
Solyc09g065100.2*	0.62	1.34	bHLH150
Solyc12g007070.2	2.62	1.28	Heat stress transcription factor C-1
Solyc11g017470.2*	2.39	1.02	NAC domain-containing protein 2-like
Solyc10g079050.2	1.46	1.71	Transcription factor bHLH130-like
Solyc10g009550.3	−2.50	−1.06	Probable WRKY transcription factor 30
Solyc09g014990.3	−1.93	−1.18	Probable WRKY transcription factor 26
Solyc06g051260.3*	−1.46	−1.25	Transcription factor bHLH51
Solyc03g115850.3	−0.99	−1.63	NAC domain-containing protein 100
Solyc10g080030.2	−1.89	−1.69	MADS-box transcription factor 23-like
Solyc12g014140.2	−1.93	−1.81	TCP transcription factor 3
Solyc11g028020.2	−1.35	−2.09	TAGL11 transcription factor
Solyc10g005010.3	−2.31	−3.38	NAC domain-containing protein 43
Solyc08g080490.3	−1.52	−1.53	2S sulfur-rich seed storage protein 2-like
Solyc09g025210.3	−1.68	−2.32	12S seed storage protein CRA1-like
Solyc09g072560.3	−1.68	−2.55	11S globulin seed storage protein 2-like

### Change in the ripening-related metabolic processes in *SlMYB75*-OE tomatoes

**Fig. 4:**
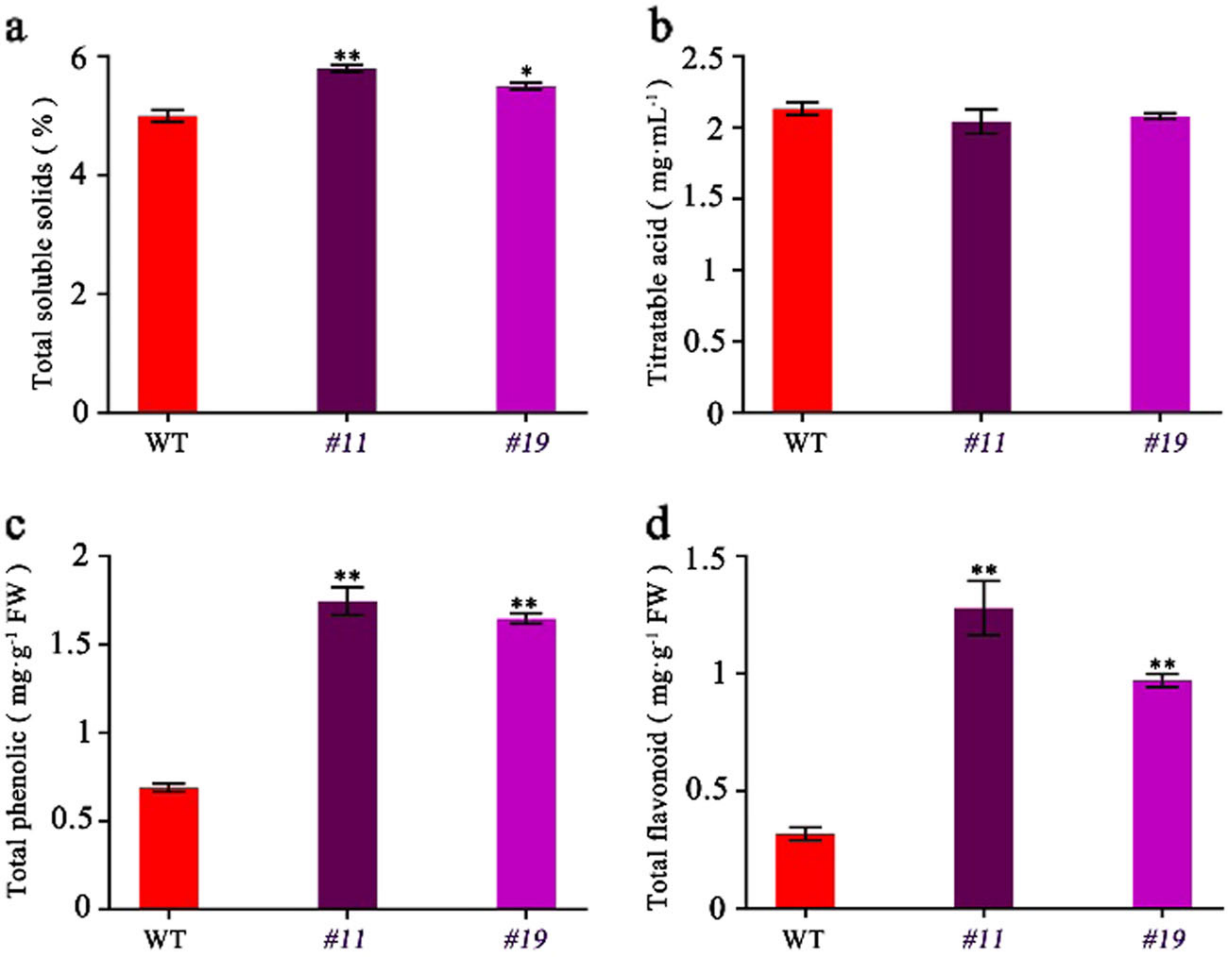
Determination of the total soluble solids, titratable acids, total phenolics and total flavonoids in WT and *SlMYB75*-OE fruits at BR + 7. Total soluble solids (**a**) and titratable acids (**b**) were measured using a digital refractometer and a titration method, respectively. The data are the means ± SD of at least 10 individual fruits for each line. The total phenolics (**c**) and total flavonoids (**d**) were calculated according to the gallic acid and rutin equivalents, respectively. All data are the means ± SD of three biological replicates, *P < 0.05; and **P < 0.01 (Student’s *t*-test)

**Fig. 5:**
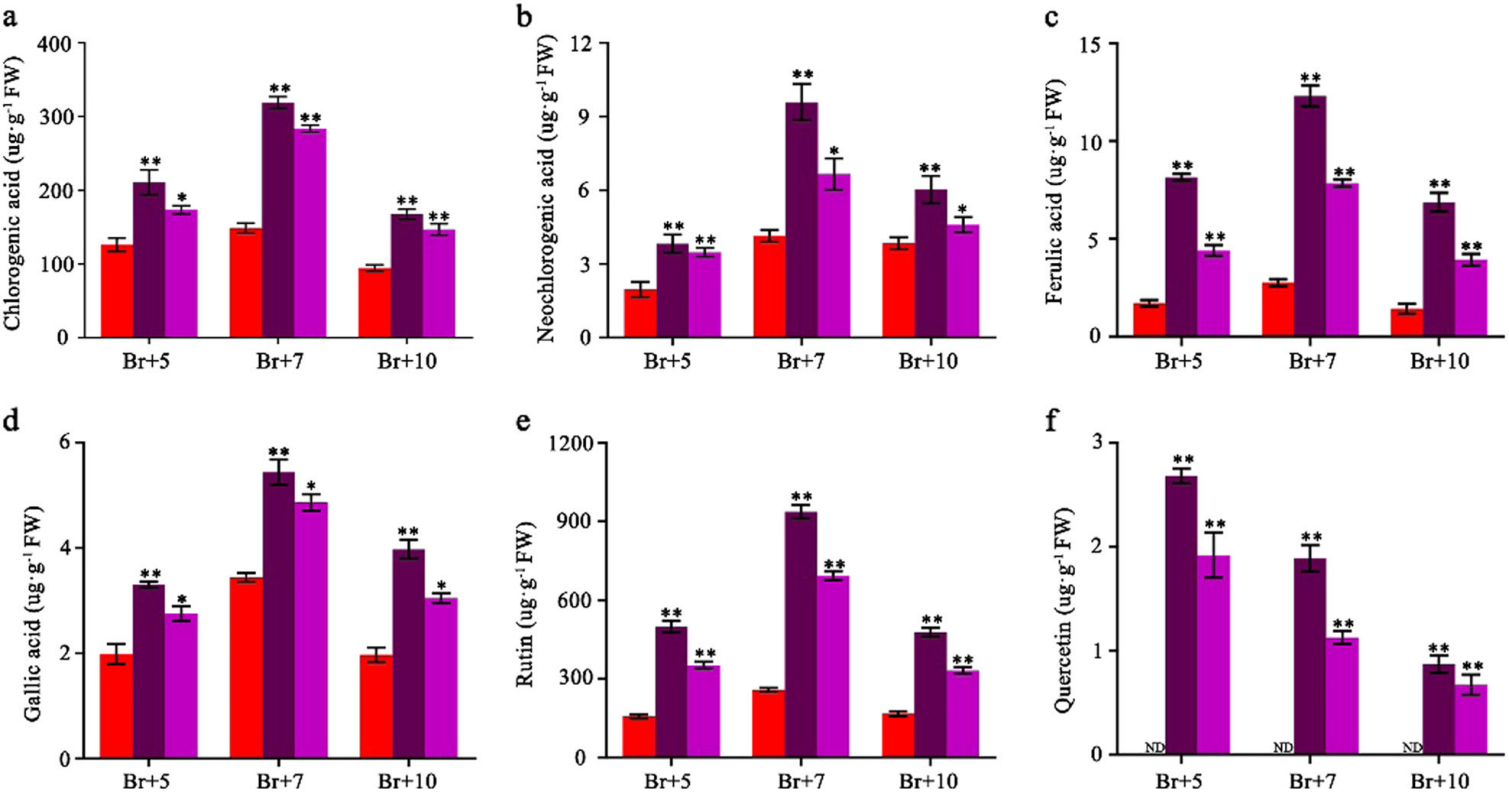
Phenolic and flavonoid accumulation in WT and *SlMYB75*-OE fruits at BR + 5, BR + 7, BR + 10. Chlorogenic acid (**a**), neochlorogenic acid (**b**), ferulic acid (**c**), gallic acid (**d**), rutin (**e**), and quercetin (**d**) were quantified according to their corresponding standards using an HPLC method. The data are the means ± SD of three biological replicates analyzed by Student’s *t*-test; *P < 0.05; and **P < 0.01

To further investigate the metabolic changes in the *SlMYB75*-OE tomatoes, we measured the metabolite contents relevant to the DEGs at the BR + 5, BR + 7 and BR + 10 ripening stages. Interestingly, the total soluble solids (TSS), which primarily reflect the sugar content, was significantly higher in the *SlMYB75*-OE tomatoes than in the WT (Fig. 4a) while the titratable acids showed no significant difference between WT and *SlMYB75*-OE tomatoes (Fig. 4b). Strikingly, the total contents of phenolics and flavonoids were dramatically increased in *SlMYB75*-OE tomatoes (Fig. 4c, d). A further characterization of the changes in phenolics and flavonoids contents revealed that the *SlMYB75*-OE fruits exhibited higher levels of chlorogenic acid, neochlorogenic acid, ferulic acid, gallic acid, rutin and quercetin than those in the WT (Fig. 5). These data indicated that in addition to increasing the anthocyanin content, overexpression of *SlMYB75* also led to elevated levels of sugar, phenolic and flavonoid compounds.

### Overexpression of *SlMYB75* affects volatile aroma metabolism

Considering that some of the DEGs such as *LOX, AADC* and *CXE1* have been reported to play critical roles in aroma volatile production^33,34^, we performed a comparative analysis of aroma volatiles in WT and *SlMYB75*-OE tomatoes. Nearly 380 volatile compounds were identified in the Micro-Tom tomato fruits analyzed in this experiment, and only 38 characteristic volatiles were screened and quantified (Table 2). Based on the chemical nature of their precursors, tomato volatiles could be categorized into the following three groups: those derived from fatty acids, carotenoids and amino acids^34^. As shown in Table 2, the aroma compounds derived from fatty acids, especially aldehydes, accounted for a large part of the tomato aroma content. The amount of aroma volatiles in fruits at the BR + 5 stage was much lower than the levels at the BR + 7 and BR + 10 stages, and some of the aroma volatiles could not even be detected at the early ripening stage. Most of the volatiles showed significantly higher levels in *SlMYB75*-OE tomatoes than those in the WT, including trans-2-hexenal, hexanal, 3-hexenal, benzaldehyde, 3-nonanone, methyl salicylate and geranyl acetate. By contrast, some volatiles such as 2-propyl-1-pentanol and 2-ethylhexyl acrylate showed similar or lower contents in *SlMYB75*-OE tomatoes compared to the WT. Noticeably, the contents of terpenoids/terpenes, terpineol, (-)-4-terpineol, copaene, γ-terpinen and β-myrcene in the *SlMYB75*-OE fruits exhibited a dramatic increase, with some of them having more than 10 times higher levels than those in the WT, while the content in the WT stayed very low. Overall, the general trend is that volatiles in *SlMYB75*-OE tomatoes are significantly higher than they are in the WT, in particular those corresponding to aldehydes and terpenes.

**Table 2:** Aroma volatiles emitted by WT and *SlMYB75-*OE fruits at the BR + 5, BR + 7 and BR + 10 stages. The data are expressed as the means ± standard deviation of triplicate samples

Volatile compound	RT (min)	BR+5 Content (ng g^−1^ FW h^−1^)			BR+7 Content (ng g^−1^ FW h^−1^)			BR+10 Content (ng g^−1^ FW h^−1^)		
		WT	#11	#19	WT	#11	#19	WT	#11	#19
Aldehydes										
trans-2-Hexenal	4.90	259.65 ± 22.48	421.29 ± 31.71	331.96 ± 22.38	193.00 ± 32.87	368.42 ± 50.84	257.05 ± 36.45	219.73 ± 35.18	352.31 ± 25.68	303.40 ± 25.90
Hexanal	3.66	77.29 ± 11.74	116.96 ± 22.90	97.84 ± 7.12	44.35 ± 11.82	89.52 ± 21.54	77.92 ± 5.11	74.98 ± 8.37	196.91 ± 46.12	128.03 ± 19.14
3-Hexenal	3.62	33.29 ± 2.98	56.18 ± 3.15	46.63 ± 2.37	12.07 ± 0.96	42.74 ± 2.73	28.51 ± 2.64	25.33 ± 2.89	48.25 ± 2.19	39.32 ± 3.28
(Z)-2-Heptenal	8.01	4.83 ± 0.54	11.46 ± 1.66	7.80 ± 0.61	6.38 ± 1.00	11.21 ± 1.80	8.86 ± 0.35	6.63 ± 0.52	14.43 ± 2.11	10.62 ± 0.74
(E,E)-2,4-Heptadienal	9.06	3.85 ± 0.24	7.64 ± 0.73	6.08 ± 0.13	2.95 ± 0.54	6.43 ± 1.76	4.77 ± 0.19	6.42 ± 1.72	16.48 ± 1.28	9.01 ± 1.47
Heptanal	6.55	2.59 ± 0.39	5.55 ± 0.70	4.97 ± 0.25	1.90 ± 0.52	4.82 ± 1.07	3.42 ± 0.43	3.16 ± 0.61	5.61 ± 1.75	4.38 ± 1.32
Geranial	15.05	12.27 ± 2.42	31.05 ± 2.99	21.22 ± 2.96	47.15 ± 7.63	276.86 ± 44.54	204.11 ± 41.07	66.92 ± 11.91	252.34 ± 15.73	179.60 ± 31.54
Neral	14.52	ND	6.33 ± 2.33	4.27 ± 1.03	ND	5.45 ± 0.46	3.18 ± 0.71	5.70 ± 1.37	22.40 ± 2.62	15.37 ± 1.96
Benzaldehyde	7.90	3.49 ± 0.89	6.38 ± 1.63	4.91 ± 1.10	3.73 ± 0.79	8.70 ± 1.13	6.70 ± 0.68	4.23 ± 0.92	7.63 ± 1.42	6.46 ± 0.45
Alcohols										
6-Methyl-5-hepten-2-ol	9.30	9.63 ± 2.62	11.04 ± 2.40	10.23 ± 0.43	19.38 ± 8.69	23.81 ± 2.63	21.54 ± 4.15	16.45 ± 3.52	27.56 ± 4.53	24.25 ± 2.73
2-Propyl-1-pentanol	10.26	20.01 ± 5.13	10.04 ± 0.54	14.64 ± 2.68	13.82 ± 2.93	5.60 ± 3.16	8.75 ± 1.57	3.36 ± 1.25	1.91 ± 0.37	2.22 ± 0.75
Eucalyptol	14.91	ND	ND	ND	ND	108.95 ± 23.39	70.36 ± 9.40	ND	85.87 ± 7.43	53.91 ± 5.61
1-Octanol	11.23	4.23 ± 0.82	9.57 ± 1.00	6.20 ± 0.34	4.71 ± 1.16	10.07 ± 2.78	7.21 ± 0.77	5.16 ± 0.49	9.60 ± 1.30	6.73 ± 0.72
Matsutake alcohol	8.98	ND	ND	ND	ND	21.52 ± 2.68	14.39 ± 2.82	ND	13.66 ± 1.27	7.89 ± 0.48
Ketones										
β-ionone	18.81	1.66 ± 0.12	1.88 ± 0.43	1.79 ± 0.29	1.77 ± 0.39	2.86 ± 0.64	2.19 ± 0.91	1.89 ± 0.59	2.78 ± 0.52	2.32 ± 0.31
6-Methyl-5-h eptene-2-one	8.96	64.57 ± 14.54	71.89 ± 18.57	69.16 ± 15.53	50.77 ± 7.89	58.18 ± 13.71	56.33 ± 12.84	161.80 ± 12.71	170.58 ± 36.07	165.23 ± 13.39
Geranylacetone	18.27	8.46 ± 1.52	15.84 ± 2.14	13.73 ± 1.92	13.82 ± 1.15	39.76 ± 5.49	27.06 ± 1.15	24.37 ± 4.34	40.88 ± 8.84	31.89 ± 5.01
3-Nonanone	11.49	2.88 ± 0.73	13.47 ± 2.38	8.18 ± 0.97	91.51 ± 0.40	202.60 ± 37.01	151.44 ± 22.51	140.37 ± 13.72	279.56 ± 24.59	216.84 ± 24.57
Farnesyl acetone	24.82	3.27 ± 0.30	5.35 ± 0.28	4.42 ± 0.18	4.35 ± 0.76	8.42 ± 0.84	7.09 ± 0.92	4.32 ± 0.71	9.78 ± 2.36	7.47 ± 1.08
Esters										
Methyl salicylate	13.59	4.75 ± 1.33	13.67 ± 1.07	11.19 ± 0.61	4.01 ± 1.69	20.95 ± 1.66	12.06 ± 1.06	2.88 ± 0.45	8.96 ± 0.89	6.97 ± 0.59
Geranyl acetate	17.15	ND	ND	ND	6.20 ± 1.04	31.49 ± 8.72	21.33 ± 3.07	6.78 ± 1.73	33.68 ± 4.80	21.09 ± 2.35
Nerol acetate	16.84	ND	ND	ND	4.65 ± 0.58	17.08 ± 2.55	10.57 ± 0.86	5.39 ± 1.33	16.32 ± 2.07	10.83 ± 1.61
α-terpineol acetate	16.66	ND	ND	ND	3.82 ± 0.43	21.86 ± 3.01	13.74 ± 2.75	4.95 ± 0.90	21.79 ± 5.01	15.64 ± 3.53
2-Ethylhexyl acrylate	14.52	87.61 ± 16.45	ND	ND	61.36 ± 20.21	ND	ND	ND	ND	ND
Isobutyl acetate	4.13	0.34 ± 0.05	0.54 ± 0.08	0.50 ± 0.10	0.20 ± 0.08	0.78 ± 0.08	0.55 ± 0.07	0.18 ± 0.03	0.30 ± 0.08	0.29 ± 0.06
Terpenoids/Terpenes										
Linalool	11.88	28.06 ± 6.20	134.50 ± 16.79	99.69 ± 6.14	34.47 ± 11.09	313.13 ± 40.92	234.12 ± 13.87	59.80 ± 13.92	370.44 ± 35.57	260.58 ± 38.45
Terpineol	13.68	4.48 ± 0.96	13.61 ± 3.12	11.10 ± 1.87	6.67 ± 3.57	27.87 ± 3.05	17.77 ± 5.06	8.62 ± 2.00	19.03 ± 4.04	12.59 ± 2.01
(-)-4-Terpineol	13.46	1.02 ± 0.46	11.08 ± 0.99	7.22 ± 0.47	16.37 ± 0.38	92.76 ± 14.58	69.05 ± 19.86	18.34 ± 3.24	63.52 ± 5.78	49.16 ± 8.37
D-Limonene	10.36	9.71 ± 1.38	19.62 ± 4.13	14.77 ± 1.53	19.79 ± 4.63	60.74 ± 14.19	16.18 ± 5.76	34.08 ± 6.72	79.74 ± 12.15	74.22 ± 9.23
β-myrcene	9.46	1.91 ± 1.05	4.38 ± 1.02	3.43 ± 1.19	12.53 ± 2.52	44.03 ± 12.17	25.94 ± 5.84	26.07 ± 2.44	50.97 ± 7.38	44.39 ± 4.12
Copaene	17.39	10.92 ± 2.15	29.06 ± 5.95	21.38 ± 2.57	4.14 ± 0.93	38.62 ± 9.59	23.16 ± 4.24	7.20 ± 1.94	9.03 ± 1.23	7.37 ± 0.57
Ocimene	10.83	0.46 ± 0.07	0.73 ± 0.50	0.58 ± 0.03	16.55 ± 2.42	24.04 ± 4.16	19.50 ± 3.43	14.59 ± 1.47	21.05 ± 3.46	16.23 ± 4.08
β-trans-ocimene	10.57	0.42 ± 0.07	1.24 ± 0.37	0.87 ± 0.13	5.41 ± 0.47	18.24 ± 1.73	12.02 ± 1.00	6.28 ± 0.90	15.26 ± 3.26	11.97 ± 2.47
γ-terpinen	11.05	0.56 ± 0.03	1.50 ± 0.25	0.92 ± 0.15	0.45 ± 0.25	7.28 ± 1.26	5.09 ± 0.96	1.45 ± 1.21	8.39 ± 0.60	6.26 ± 0.16
Terpilene	10.06	ND	ND	ND	ND	5.71 ± 0.50	3.09 ± 0.35	ND	6.18 ± 0.53	3.62 ± 0.45
Terpinolen	11.72	ND	1.12 ± 0.15	ND	ND	7.12 ± 2.15	3.21 ± 0.59	1.60 ± 0.23	11.88 ± 1.61	6.84 ± 1.81
α-phellandrene	9.74	ND	ND	ND	ND	5.10 ± 0.98	2.97 ± 0.69	3.82 ± 0.58	6.48 ± 0.93	4.96 ± 0.36
β-cyclocitral	14.15	1.02 ± 0.25	1.99 ± 0.23	1.56 ± 0.20	1.30 ± 0.30	1.84 ± 0.14	1.52 ± 0.35	1.35 ± 0.48	2.68 ± 0.51	2.08 ± 0.38

*Note*: The data are expressed as the means ± standard deviation of triplicate samples. *RT* retention time, *ND* not detectable

### Analysis of the regulatory relationships between SlMYB75 TF and other genes

**Fig. 6:**
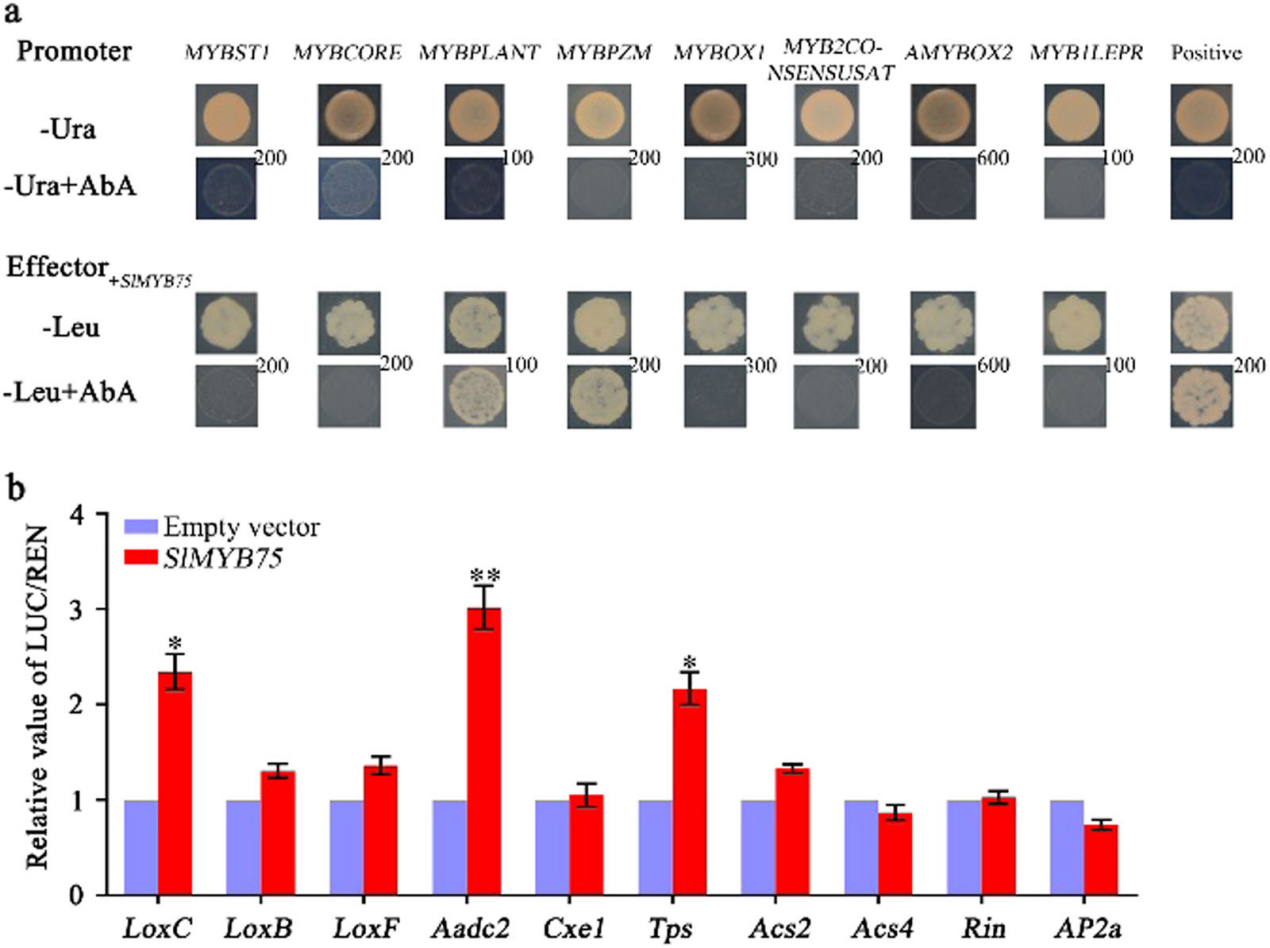
Regulatory roles of SlMYB75 on the conserved *cis*-elements and promoters of several DEGs. **a**, The interactions between SlMYB75 and eight conserved *cis*-elements were determined by the yeast one-hybrid assay. No auto-activation ability was detected in the eight conserved *cis*-elements in yeast grown on SD/ura medium containing aureobasidin A (-Ura + AbA). The interactions were determined on SD/leu medium in the presence of AbA (-Leu + AbA). **b**, The transcription activation ability of SlMYB75 on the promoters of several DEGs was tested using dual luciferase assays. The LUC/REN ratio of the empty vector plus the promoter was used as the calibrator (set as 1). At least six biological replicates were conducted for each combination. The values are the means ± SD. *P < 0.05; and **P < 0.01 (Student’s *t*-test)

To explore whether DEGs could serve as direct targets of SlMYB75, we checked the ability of this MYB TF to bind the promoters of some of the DEGs directly using a yeast one-hybrid (Y1H) assay. Since many DEGs were identified in this study, we chose highly conserved elements rather than specific DEGs to verify which ones could be directly bound by SlMYB75. By analyzing the promoters with the New Place database tools, we identified eight conserved elements as the primary ones present in the DEG promoters (Supplementary Table S1). Tests on these eight conserved elements in Y1H assays indicated that only MYBPLANT (AAACCAACCC) and MYBPZM (ACCTACCC) could be recognized by SlMYB75 (Fig. 6a), suggesting they might be direct targets of this MYB gene. To verify whether SlMYB75 could interact with native promoters of some DEGs, we performed dual-luciferase assays with genes involved in ethylene signaling and volatile aroma metabolism. The data showed that SlMYB75 could only trans-activate the promoters of the *LOXC*, *AADC2* and *TPS* genes (Fig. 6b).

## Discussion

In the last period, both classical and biotechnology-based breeding methods have made major progress that opened new prospects for crop improvement^3,18,35^. Anthocyanins are well known for their strong antioxidant and health-protection properties, but some of the most highly consumed fruits, such as tomatoes, are completely devoid of this compound. In the present study, we obtained remarkable purple tomatoes enriched with this health-promoting compound through the ectopic expression of a single SlMYB75 TF. Strikingly, *SlMYB75*-OE fruits also exhibited higher phenolics and flavonoids contents and enhanced the production of aroma volatiles. Our results indicated that SlMYB75 TF plays excellent roles in promoting fruit quality, and it can be directly used in genetic engineering or traditional breeding methods to produce high quality fruit.

The purple phenotype of *SlMYB75*-OE fruits in this study is remarkable, and its anthocyanin content could increase to 1.86 mg g^−1^ fresh weight (Fig. 2), which exceeded the results of earlier studies through overexpression of a single gene and previously required at least two genes to reach this level^3,13,14,18,23,24^. Given that it is difficult to find the parental lines that could highly express these two or three genes at the same time, together with its easy segregation characteristic, multi-gene strategies are not the best choice for breeders. Thus, expressing a single *SlMYB75* provides a straightforward strategy towards improving the fruit nutritional and sensory quality. In addition, the content of phenolic and flavonoid compounds was also greatly enhanced in SlMYB75-OE fruits, which is similar to the results of a study on anthocyanin-producing grapes^36^. Phenolics, flavonoids, and anthocyains are all derived from phenylalanine, and the transcriptomic data showed that most of the genes involved in the phenylalanine pathway were upregulated in *SlMYB75*-OE fruits. Thus, *SlMYB75* might participate in regulating different branches of the phenylpropanoid pathway.

It has been shown that there was no difference in the seed sizes between the WT and *SlAN2*-expression lines^24^. However, our results indicated the seeds of *SlMYB75*-OE tomato were significantly smaller than the WT ones (Fig. 1d). Previous studies indicated that plants that produce small seeds usually exhibit superior colonization abilities^37^, and thus *SlMYB75*-OE tomatoes may have a better dispersal capacity due to their abundant small seeds. In addition, it has been reported that *SlAN2* overexpression confers enhanced tolerance to abiotic stresses such as cold, high temperature, high light, and oxidative stress^21–23^. This finding is consistent with our data showing that *SlMYB75* is also responsive to NaCl, ABA, and SA (Supplementary Fig. S2). Collectively, these traits might confer a better capacity to survive under harsh environmental conditions to *SlMYB75*-OE tomatoes.

While ethylene is known to regulate fruit ripening positively through inducing ripening-associated genes and autocatalytic ethylene production^38^, two seemingly conflicting features of *SlMYB75*-OE tomatoes are their high level of ethylene production and delayed ripening (Fig. 2). As an attempt to explain the two seemingly inconsistent features, it may be speculated that the higher anti-oxidative properties of *SlMYB75*-OE tomatoes could mitigate the ethylene effect, since the ripening process comprises a series of physiological and biochemical changes, among which the anti-oxidative characteristics have been suggested to play a fundamental role^39,40^.

Aroma volatiles play a key role in the perception and acceptability of flowers, vegetables and fruits by consumers^34^. This property leads aroma towards being the focus of horticultural research, but little is known about improving the aroma content through the transcriptional regulation of fruits^41^. Consistent with the study in petunia flowers^42^, our results showed that the contents of the phenylalanine-derived aroma volatiles benzaldehyde and methyl salicylate were significantly higher in *SlMYB75*-OE fruits. Furthermore, the transcript level of fatty acid-related genes (*LOXC*, *LOXB* and *LOXF*)^43^ and the content of most fatty acid-derived aroma volatiles, especially trans-2-hexenal and (z)-2-heptenal, which are important contributors to flavor and consumer acceptance of tomato fruits^44^, were greatly increased in *SlMYB75*-OE fruits. The primary contributors to aroma volatiles in blood oranges ranked from high to low are as follows: terpenes, esters and then aldehydes^45–47^, but tomato fruits have very low levels of terpenes and contain only minute amounts of accumulated monoterpenes^48^. Interestingly, the terpene levels (Table 2) and transcript level of a terpene synthase gene (*Solyc06g060180.2*) (Table 1) were also greatly upregulated in *SlMYB75*-OE tomato fruits, suggesting that the isoprenoid pathway was also affected in *SlMYB75*-OE tomatoes. Studies on tomato mutants and rice indicated that there are interactions between the phenylpropanoid and isoprenoid pathways, but the mechanisms underlying the interactions responsible for the production of specific metabolites are still unclear^49,50^. To explore the molecular mechanism of *SlMYB75* in promoting aroma volatiles, yeast one-hybrid and dual-luciferase assays were performed. The yeast one-hybrid assays revealed positive interactions between SlMYB75 and conserved MYBPLANT (AAACCAACCC) and MYBPZM (ACCTACCC) elements, supporting the idea that the CC(T/A)ACC sequence motif might be the core binding site of SlMYB75 (Fig. 6a), in a similar way with that of AtMYB12 as reported previously^18^. Consistent with this finding, the dual-luciferase assays showed that *LOXC*, *AADC2* and *TPS* genes with promoters containing MYBPLANT (AAACCAACCC) and MYBPZM (ACCTACCC) elements (Supplementary Fig. S5) can be directly trans-activated by SlMYB75 (Fig. 6b). Thus, SlMYB75 has a regulatory function and promotes aroma volatile accumulation, probably by targeting downstream aroma volatile-related genes.

Overall, the outcome of the study indicates that overexpression of a single SlMYB75 TF can improve several tomato quality traits, including the sensory and nutritional aspects. This finding provides new opportunities for innovative breeding strategies aimed at generating tomato lines that better meet consumer requirement in terms of enriching health-promoting metabolites while better coping with a changing environment.

## Electronic supplementary material

Supplementary material

File S1

File S2

File S3

File S4

Supporting information
