# Supplementary material for: SlMYB75, an MYB-type transcription factor, promotes anthocyanin accumulation and enhances volatile aroma production in tomato fruits
Source: Hortic Res. 2019 Feb 1;6:22. doi: 10.1038/s41438-018-0098-y (PMC6355774; doi:10.1038/s41438-018-0098-y)
Supplement: Supplementary file 1 — Supplementary material [file 41438_2018_98_MOESM1_ESM.pdf]

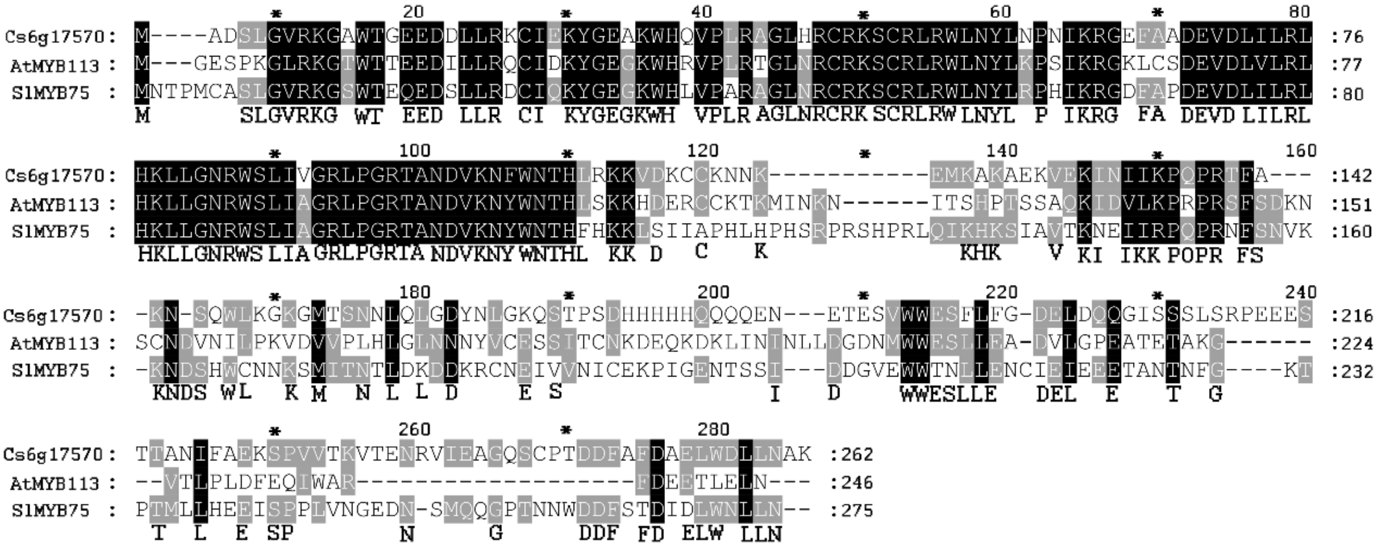

Supplementary Fig S1. Alignment of amino acids specific to Cs6g17570, AtMYB113 and SlMYB75. The amino acids were aligned using CLUSTAL X and analyzed with MEGA version 6 software. Completely conserved and highly conserved residues are colored black and grey, respectively. Dashes are gaps introduced to maximize alignment. The alignment was produced from three amino acid sequences from *Citrus sinensis (*Cs6g17570*, KT757348), Arabidopsis thaliana (*AtMYB113*, NM_105308), Solanum lycopersicum (*SlMYB75*, solyc10g086250)*.

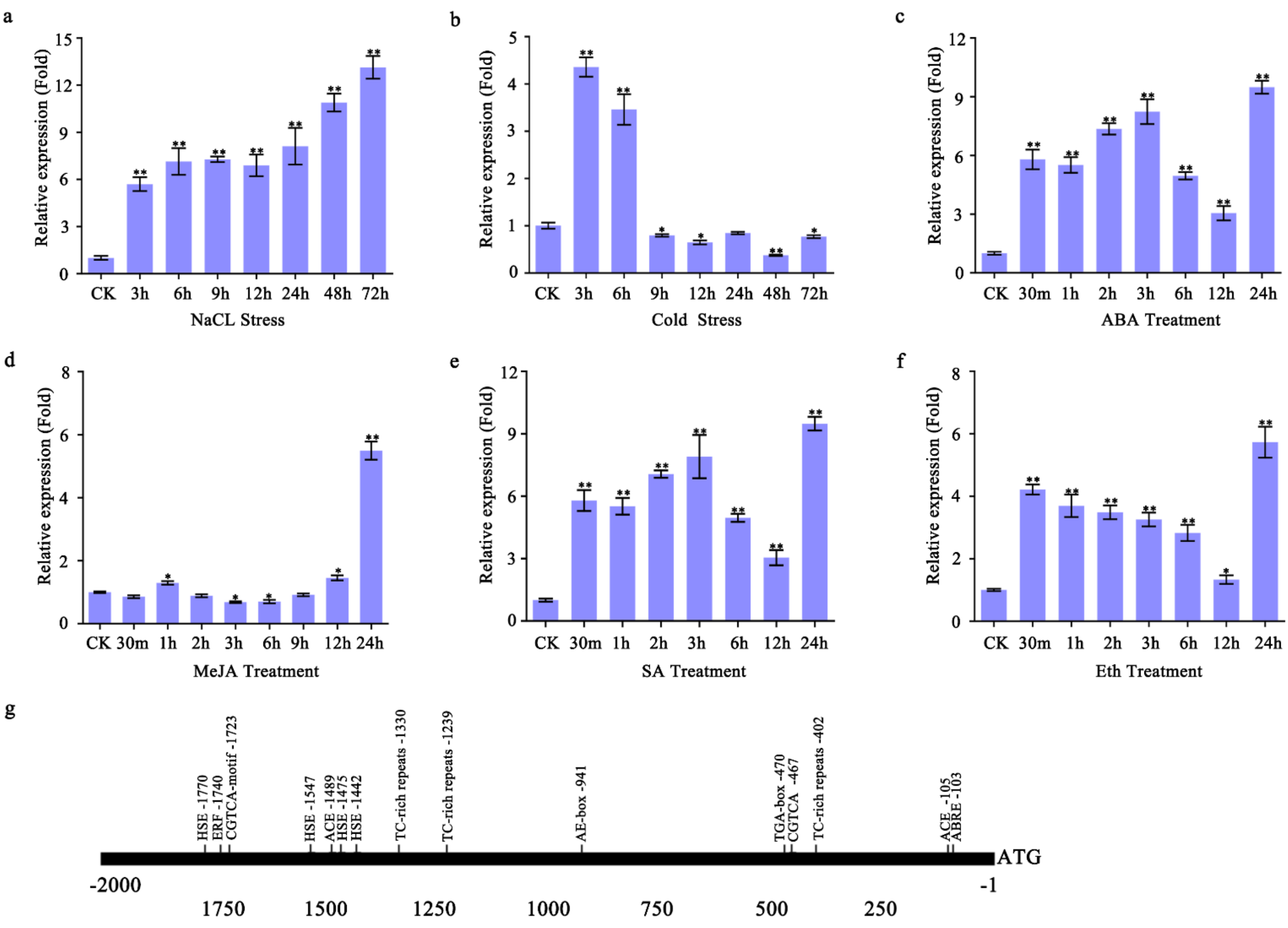

Supplementary Fig S2. *SlMYB75* was involved in various hormones or stresses response. **a**-**f**. Leaves of one-month-old tomato plants treated with NaCL, Cold, ABA, MeJA, SA and Eth were harvested to analyse the gene expression level. Data represent means ± SD of three biological replicates and analysed by student's t test. *, P <0.05; and **, P <0.01 (Student's t test). **g**, Promoter region of *SlMYB75* and its putative cis-elements.

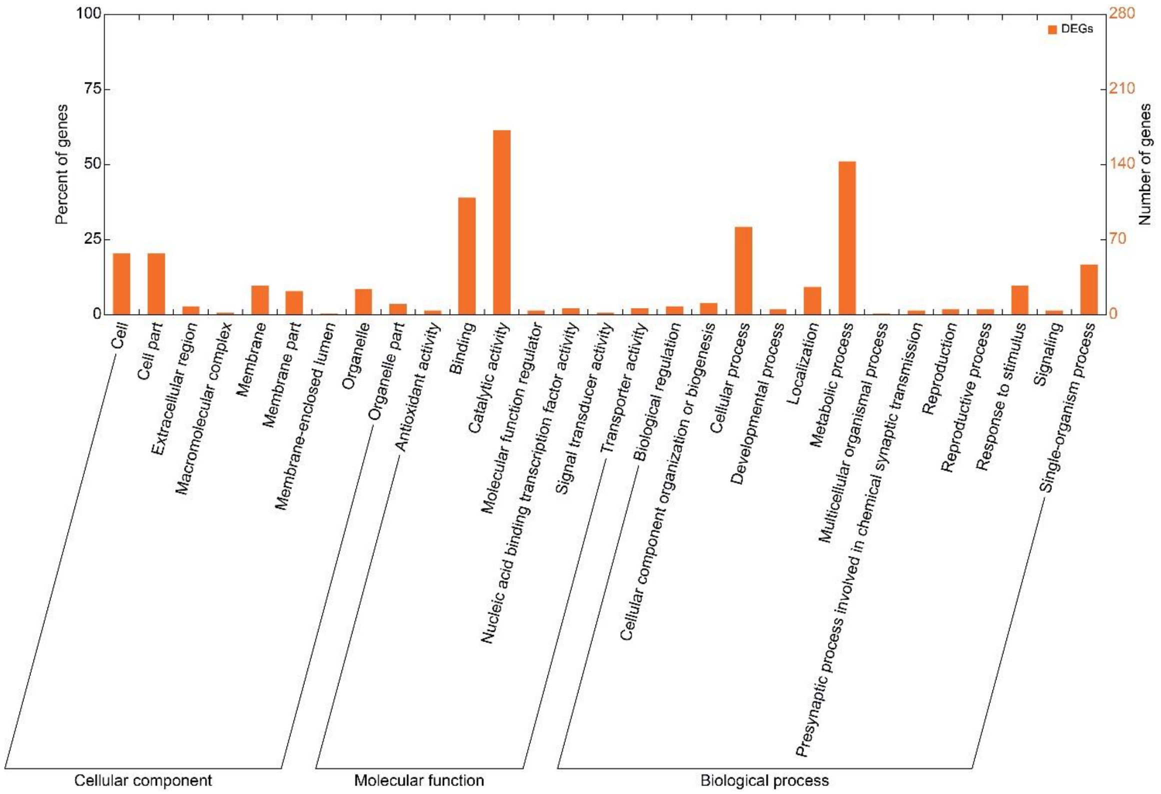

Supplementary Fig S3. Gene ontoloty (GO) enrichment analysis of the DEGs with predicted function. The go terms enriched by DEGs based on biological process, cellular component and molecular function, respectively.

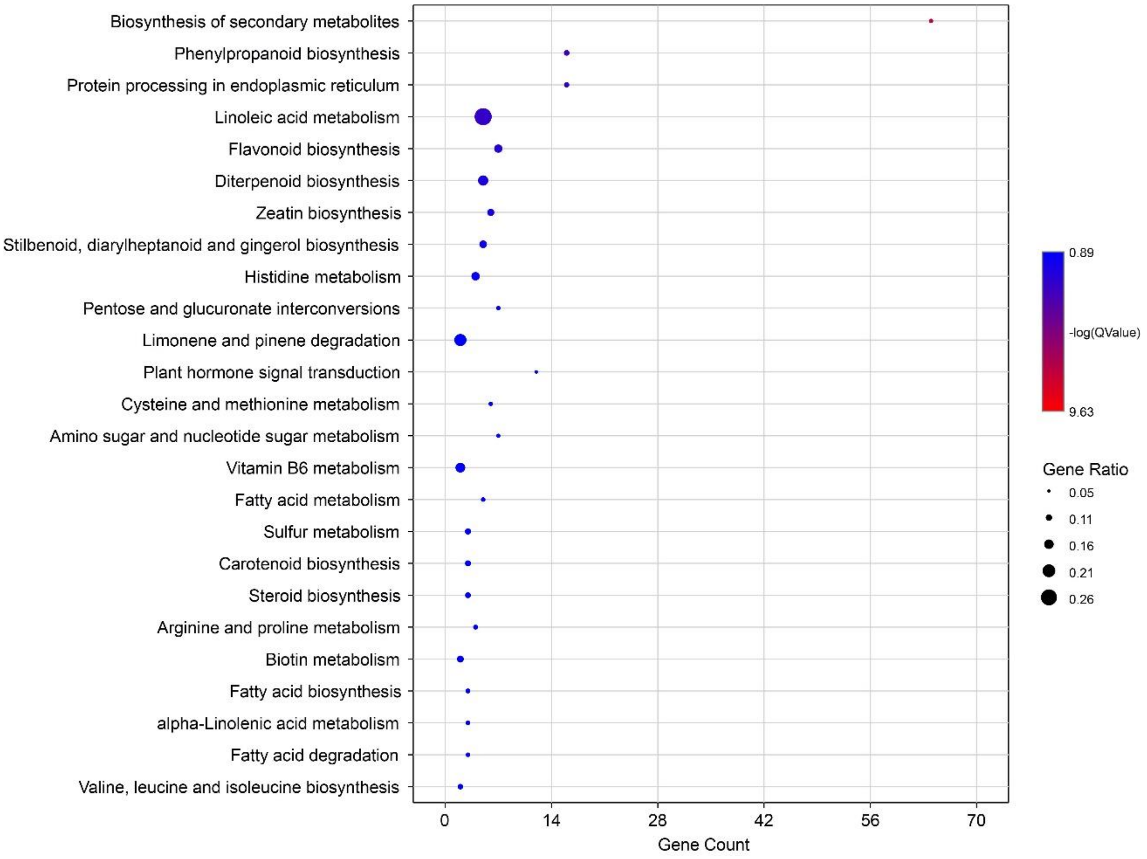

Supplementary Fig S4. KEGG enrichment scatter plot of DEGs. The y-axis represents the name of pathway, and the x-axis represents the gene count. The color represents the –log (q-value) and the dot size represents the gene ratio.

Supplementary Table S1 Information of eight MYB-binding conserved *cis*-elements used in yeast one-hybrid experiments.

**Conserved Element**	**Sequence**	**Representative sequence**
MYBST1	GGATA	TGGATAA
MYBCORE	CNGTTR	TCAGTTAT
MYBPLANT	MACCWAMC	AAACCAACCC
MYBPZM	CCWACC	ACCTACCC
AMYBOX1	TAACARA	ATAACAAAT
MYB2CONSENSUSAT	YAACKG	TTAACTGA
AMYBOX2	TATCCAT	ATATCCATG
MYB1LEPR	GTTAGTT	AGTTAGTTG

a

TGAAGAGTTAATATTTAGTGAAAATTGTGGGCCAGCTTGAATGAAAACTTTGATATTTGTTTGATAAAAAAACACGTATTAATGCTGATTTAT
TTTTAAAATATTTGATAACTGAGTAATTTTTTTTATATATAATATTTATTAATATAACAAAATAAATATAACGTTGGGTAAAATTATCAAATCTAAA
TTAATTTATATTTATTTTAAATAGATTTAAAGAATGACTATAAGAAAGTAGAAATAACGAAGATAGACATAATATCATAAACCAAGGAAAATGA
GTATTATAGAGCGAAATCGAATGAAAGTCTCTAAAATTATCCCTTTTATTTTATACATTAAAAAAAATCTGAAAATAGTTAAAGAGATCTCAAT
TTAGCTTTAACACATTAAGAAAATTATTTATTATCCACCAAGTGAAAAATATTTCTAGGACGCAATTTATTGTCTCCTTAAGGCAAAATTATTT
TATTAAAAAAGCTATTAATTAATAGGTATAAATAGTAATTTAGATTACAACAACTCTTAAGATAAAGAATTCTGAAATCATAAGCTGTTTTTTTC
CTGTCAATTTATGATTGCTATATATTGTATTTATTCAATTTTAAGTGTCACAACTTTTGACAACATTTTTATTTTTCAACATATATACCTCAATAAT
ATAAGTTTTCTGCGAATTTTCAGACTAATCCCAAAGAAAAAATTCTCATGTAATTCACACTTATTTTTATAAAAATCCATTATAAATATGCATAC
ATATTTATAAATTGTGAAACTCAGTAATTAAAACGAGCGATTAATTTGATATAAGTTCAGAACTCATAAACTTTAAATTCCGATTCAGCGTTCG
ATAATATCTTGAAAAAAATTCTACATGCCCAAAACAATTCACACACATAGGTACCTAAGCAAAACTTCATCATCTATATAAAGAGAACTCCTTA
AGAGACCAAAATGCA**AACCAAAC**CATATATCAATTAAAGTTTGAAATAATAAAA

b

TAATAGTTATTGATTTTATTAATGAGTTGAAGTCTTCCGCATTACTTTATGTTGATATTATATTGAAATGTTAAGGTTTAGATT**GGTTGGTT**CGC
TCACATAGGAGGGTAAGTGTGGGTGCCAGTCGCGGCTCGGATTTGGGTCGTGACACAAAAAGAGGATTTTTACCTCACATAAAATAAAAG
ATCGATAAAGTCTATGACTATGCATATACGTCACATTTACCAGACCTAACTTATGAAATTTCATTATTAATGTTTACGACCTCTATGTTTAGAAA
CCACTTGTGCCATGAATCTTTGAATTGGTTTAGAAACCACTTGACAAATAATGGTACACTAATAACGTATCAAATTCCATATAATTTATAGTTT
CTCAAAATTGATAAAACTATTATAACAAACACAAAATGAATCACTTAACATTGTCGAGTTTCTTCTTGAGTCAAGGGTTTATCATAAAGAACT
TTTTTACCTCACGTAAGATAGAGATAATATCTTCGACTCTACATATACCTAACAATTATCGAACCTGACTTATGAAATTAATTTCACAGAATATG
TTATTGCTGTTTTAGAAAGTATAAGTCTAAAAACAACTTTGTGCATGAATCTTTGAATTGGAGATTACTATTCT**CACCTAAC**ATTTTTGCCAAA
GTTTGAGAAGTCTTTGTAACACAGAGTCAAAGAGTTATACTTTGTGCAAAAATTATGACTTATTTTTAATTTTAATAAATTTTTTTATTTTAGT
GGGAAAACAACATAAAGAAGGAAACAAATAGGAAAAGGTGAGGAAATAAGAAAACAAAAAATAAAAGTGAGTTGT**GGTTGGTG**CCCA
AACCCACAATCTTTTCTACCTATATATAATCCAAGAATATTATACATAAATTATATCCAAACTTGATTTTCCATTCTAAAACTAACCAAAAAAATA
AAAAATTTAGCTCCAAGA

c

TAATATTTTCAAGAAAAAAAATTTAAGATTGACGGGAATAAGCGAAATTCCTGACTTTTTACCCGTCAAACTTCTTTGAAAGTAATTTCACAC
GTGCTTTCTCGTAATTCTGAAGCAATCTCCAAAAATAGATCAAACTTAAAAACACAATTGAGTTATAAATAAAAATTCAACTAAAGAACATG
ATAATACATTTTTTTTTAAAAAATAACTCATTTGGAGATTTTCAAATAATTATAAAAATATATTTAATCAAATTCTTCAAAAGTGTCGGGTTCTT
CAAAATTAGTATTAATTTAAAAAATTCCATATAAGTACGAGATTTCGCCGGTAAAAAATCCGGGAAGTCCGCATATTGCCGCTAGTCTTATTT
TTTTCTCGAAAATATTA**GGTAGG**ATACACTTAATTATTTTATTTGATAGTTTTAAAAAATAAAAACAATTGATGATTTTGTTGTTTTTATTATTAT
AATGAGTATATAATTTAAACAGATCTCAACCACAAGTTATTATACAATACACATGACATGAATCTATAAGGTCATTAAACATTACTTGATCGGAT
CTTTATCCATCGTACCCCTTCGCCCAACAACTCATTTAATATGTACTAGAGAGAGTATTAGTTAGTCCACCTTCTATTTTTGCATAAAATAATAC
ATACATATATATATATATGTTCTCATAAGTCATAGCTTATACATGTATTAATTTGCTCAAATTTCTAAAGTATTAACCGAACACTGTGATAAATTTA
TATAAGAATAAATCACCTCTTAACCAACTACGGAACATAGTACTCCTTAAATACTTTACTCATACAAAATTTAATACCTGCATAATAACTTACCT
CCAAATCAATTATCAAATACTTCACTCATACAAAATCTAACACCTACATAATAACTTACGTCCAAACCAATTACCAAACGTCGATAGTACTCTTA
GTATAACAACCAAGAGATAATGAACTAGAGAGACCCTTTTTGGAGGTGTGTGTCTATATCTATATATGTGTGTGTGAGAGAG**GGTAGG**TATG
AGTGGAGCA

Supplementary Fig S5. Analysis of promoter sequence upstream of the ATG of *LOXC*, *AADC2* and *TPS* genes. The 1000bp promoter sequence of *LOXC* (**a**), 951bp promoter sequence of *AADC2* (**b**) and 1046bp promoter sequence of *TPS* (**c**) were analysed by New Place database. MYB2AT, MYBCONSENSUSAT and MYBCORE were highlighted in yellow; MYBOX1 and MYBGAHV were highlighted in green; MYB1AT was highlighted in blue; MYBST1 was highlighted in pink; MYB1LEPR was highlighted in purple; MYBATRD22 was highlighted in cyan; AMYBOX2 was highlighted in gray; MYBPLANT and MYBPZM were highlighted in bold and red.

Supplementary Table S2 Primers sequence used in amplification, qPCR, yeast one-hybrid and dual-luciferase experiments.

Name/ID	Primers Sequence	
	Forward (5'-3')	Reverse (5'-3')
SlMYB75	GCGGCCGCATGAATACTCCTATGTGTGCATCGT	CCTGCAGGATTAAGTAGATTCCATAAGTCAATA
q-SlMYB75	CCTCAACCTCGGAACTTCTC	TCTCCTATTGGCTTCTCACAAA
q-SlUBI	GCCGACTACAACATCCAGAAGG	TGCAACACAGCGAGCTTAACC
SlPAL/Solyc00g282510.2	AATTGCTTCTAGTCGTGGACAG	ACAAGGACTTGTCTCAGCTTCTG
SlANS/Solyc08g080040.3	ACGAACAGGATTTTGCTGCT	TTTGAGCTCAGCAACTGCAT
CHS1/Solyc09g091510.3	ACCAACAAGGTTGCTTTGCC	GAGATTCACTGGGTCCACGG
3GT/Solyc10g083440.1	AAACAAGGCAATGACACGCC	CCCTGTTTCCTCCTCTGCTT
LOXC/Solyc01g006540.3	GCAATGCATCATGTGTGCTA	GTAAATGTCGAATTCCCTTCG
LOXB/Solyc01g099190.3	ACATCGTGGCATACAGGTCA	TGGATAGCCACCAGATAATTGGA
CXE1/Solyc01g108560.4	ATGCCAATGGTGATGGTCGT	ATCGGATCCTTCCCCCAGAA
TPS/Solyc06g060180.2	AAGATCTGGACCTGGCAAGC	TTGCGCGACTATACTGAGGC
ACS2/Solyc01g095080.3	TGTTAGCGTATGTATTGACAACTGG	TCATAACATAACTTCACTTTTGCATTC
ACS4/Solyc05g050010.3	CTCCTCAAATGGGGAGTACG	TTTTGTTTGCTCGCACTACG
E4/ Solyc03g111720.3	AGGGTAACAACAGCAGTAGCA	CCCAACCTCCGTCTTCAC
E8/ Solyc09g089580.3	GGCACCATTCAACATACCG	CTTTCACCGAAGAAGCACG
RIN/ Solyc05g012020.3	ATGCAGCACCATCAACACAT	CTCCAAATTCAAAGCATCCA
Bhlh150/Solyc09g065100.2	CCTCTCTTGGACGGTGTTGT	GCTTGTTGTGGCTCATTGAA
Solyc11g017470.2	CGAAACTTCACGCCGATTCC	TTCCCACTCACTCGGTTTCG
Solyc06g051260.3	TGAGATTCAGCAAGAGGGGT	CCGGTCCCACACATTCAAGA
AD-SlMYB75	CGGAATTCATGAATACTCCTATGTGTGCATCGT	CGAGCTCATTAAGTAGATTCCATAAGTCAATA
AbAi-MYBST1	CTGGATAATGGATAATGGATAAG	TCGACTTATCCATTATCCATTATCCAGAGCT
AbAi-MYBCORE	CTCAGTTATTCAGTTATTCAGTTATG	TCGACATAACTGAATAACTGAATAACTGAGAGCT
AbAi-MYBPLANT	CAAACCAACCCAAACCAACCCAAACCAACCCG	TCGACGGGTTGGTTTGGGTTGGTTTGGGTTGGTTTGAGCT
AbAi-MYBPZM	CACCTACCCACCTACCCACCTACCCG	TCGACGGGTAGGTGGGTAGGTGGGTAGGTGAGCT
AbAi-AMYBOX1	CATAACAAATATAACAAATATAACAAATG	TCGACATTTGTTATATTTGTTATATTTGTTATGAGCT
AbAi-MYB2CONSENSUSAT	CTTAACTGATTAACTGATTAACTGAG	TCGACTCAGTTAATCAGTTAATCAGTTAAGAGCT
AbAi-AMYBOX2	CATATCCATGATATCCATGATATCCATGG	TCGACCATGGATATCATGGATATCATGGATATGAGCT
AbAi-MYB1LEPR	CAGTTAGTTGAGTTAGTTGAGTTAGTTGG	TCGACCAACTAACTCAACTAACTCAACTAACTGAGCT
pGREEN62- SlMYB75	CGAGCTCATGAATACTCCTATGTGTGCATCGT	GCTCTAGATTAATTAAGTAGATTCCATAAGTCA
pGREEN0800-LOXC	CCCTCGAGTGAAGAGTTAATATTTAGTGAAAAT	GCGTCGACTTTTATTATTTCAAACTTTAATTGA
pGREEN0800-LOXB	GGGGTACCGTTATATTTTTAATTTCATTTGTTG	GCGTCGACGATTAACTATTTAAACAGAAATATT
pGREEN0800-LOXF	GGGGTACCTATATATATTATATATATATATATA	GCGTCGACTTTTTTTAAAAAAAAAATGTTTTGT
pGREEN0800-AADC2	GGGGTACCTAATAGTTATTGATTTTATTAATGA	GCGTCGACTCTTGGAGCTAAATTTTTTATTTTT
pGREEN0800-CXE1	GGGGTACCCGGACTTAATATTTTAAAATAATGG	GCGTCGACTGCTAAAAGCTAAGTTGCAAAGAAG
pGREEN0800-TPS	GGGGTACCTAATATTTTCAAGAAAAAAATTTAA	GCGTCGACTGCTCCACTCATACCTACCCTCTCT
pGREEN0800-ACS2	GGGGTACCTTGAGAAAATACTTTCTATGAAAAT	CCCTCGAGTTTTTTTTACTAAATGAGTTTAGAA
pGREEN0800-ACS4	GGGGTACCCAGTTAATAATTCAAAATATTTAAA	GCGTCGACTAGTCAAATCAATCAAAAAAAGGCC
pGREEN0800-RIN	CCCTCGAGTGTGACTATTGATAGAATCCATTCA	GCGTCGACATTGTATGAAGAAAAAAAATGTAAA
pGREEN0800-AP2a	GGGGTACCTTGGGTTTAAGAAACAAGAGGAGAA	GCGTCGACTTCTTTTGTCGATAGAATTAAAATG
