# Supplementary material for: SlMYB75, an MYB-type transcription factor, promotes anthocyanin accumulation and enhances volatile aroma production in tomato fruits
Source: Hortic Res. 2019 Feb 1;6:22. doi: 10.1038/s41438-018-0098-y (PMC6355774; doi:10.1038/s41438-018-0098-y)
Supplement: Supplementary file 6 — Supporting information [file 41438_2018_98_MOESM6_ESM.docx]

**Supporting information**

Additional Supporting Information may be found online in the supporting information tab for this article:

**Supplementary Fig S1.** Alignment of amino acids specific to Cs6g17570, AtMYB113 and SlMYB75.

**Supplementary Fig S2.** *SlMYB75* is involved in various hormone or stress responses.

**Supplementary Fig S3.** Gene Ontology (GO) enrichment analysis of the DEGs with their predicted function.

**Supplementary Fig S4.** KEGG enrichment scatter plot of DEGs.

**Supplementary Fig S5.** Analysis of the promoter sequence upstream of the ATG of the *LOXC*, *AADC2* and *TPS* genes**.**

**Supplementary Table S1.** Information on the eight MYB-binding conserved *cis*-elements used in the yeast one-hybrid experiments.

**Supplementary Table S2.** Primer sequences used in amplification, qPCR, yeast one-hybrid and dual-luciferase experiments.

**File S1.** DEGs between WT and *#11* tomato fruits at the MG stage.

**File S2.** DEGs between WT and *#11* tomato fruits at the BR+0 stage.

**File S3.** Detailed information on the Gene Ontology (GO) enrichment analysis.

**File S4.** Detailed information on the KEGG enrichment.
